# "Handbook of biomedical optics", edited by David A. Boas, Constantinos Pitris, and Nimmi Ramanujam

**DOI:** 10.1186/1475-925X-11-7

**Published:** 2012-02-10

**Authors:** Boris Gramatikov

**Affiliations:** 1 Laboratory of Ophthalmic Optics, Wilmer Eye Institute, Johns Hopkins University School of Medicine, Wilmer Rm. 233, 600 N. Wolfe St. Baltimore, MD 21287

## Abstract

David A. Boas, Constantinos Pitris, and Nimmi Ramanujam, Eds.:

Handbook of Biomedical Optics

CRC Press, Taylor and Francis Group, Boca Raton, London, New York, 2011

ISBN: 978-1-4200-9036-9 (Hardback), 787 pages

## Competing interests

The authors declare that they have no competing interests.

